# Use of QSAR Global Models and Molecular Docking for Developing New Inhibitors of c-src Tyrosine Kinase

**DOI:** 10.3390/ijms21010019

**Published:** 2019-12-18

**Authors:** Robert Ancuceanu, Bogdan Tamba, Cristina Silvia Stoicescu, Mihaela Dinu

**Affiliations:** 1 Faculty of Pharmacy, Carol Davila University of Medicine and Pharmacy, 020956 Bucharest, Romania; Robert.ancuceanu@umfcd.ro (R.A.); mihaela.dinu@umfcd.ro (M.D.); 2 Advanced Research and Development Center for Experimental Medicine (CEMEX), Grigore T. Popa, University of Medicine and Pharmacy of Iasi, 700115 Iasi, Romania; 3 Department of Chemical Thermodynamics, Institute of Physical Chemistry “Ilie Murgulescu”, 060021 Bucharest, Romania; cristina.silvia.stoicescu@gmail.com

**Keywords:** c-src-tyrosine kinase, QSAR, molecular descriptors, virtual screening, drug discovery, cancer, molecular docking

## Abstract

A prototype of a family of at least nine members, cellular Src tyrosine kinase is a therapeutically interesting target because its inhibition might be of interest not only in a number of malignancies, but also in a diverse array of conditions, from neurodegenerative pathologies to certain viral infections. Computational methods in drug discovery are considerably cheaper than conventional methods and offer opportunities of screening very large numbers of compounds in conditions that would be simply impossible within the wet lab experimental settings. We explored the use of global quantitative structure-activity relationship (QSAR) models and molecular ligand docking in the discovery of new c-src tyrosine kinase inhibitors. Using a dataset of 1038 compounds from ChEMBL database, we developed over 350 QSAR classification models. A total of 49 models with reasonably good performance were selected and the models were assembled by stacking with a simple majority vote and used for the virtual screening of over 100,000 compounds. A total of 744 compounds were predicted by at least 50% of the QSAR models as active, 147 compounds were within the applicability domain and predicted by at least 75% of the models to be active. The latter 147 compounds were submitted to molecular ligand docking using AutoDock Vina and LeDock, and 89 were predicted to be active based on the energy of binding.

## 1. Introduction

Src (c-src, pp60-src, or p60-src) is a nonreceptor, cytoplasmic tyrosine kinase, the first of its kind to be discovered (in the 1970s) in the living world, whereas the corresponding gene was the first oncogene to be uncovered [1]. It is the prototype of a larger family, comprising at least nine members, most of them with little activity in normal cells in the absence of stimulatory signals [2]. Src kinases have been suggested to be involved in the exacerbation of neurodegenerative pathologies, whereas their inhibition would diminish microgliosis and mitigate inflammation, findings that are in line with experimental effects seen for nonspecific src inhibitors such as bosutinib or LCB-03-0110 [3]. Nonclinical evidence has pointed to the inhibition of src kinases as a possible method of therapy for the pulmonary vascular remodeling and right ventricular hypertrophy in pulmonary hypertension [4], although several reports indicate that dual Abl/src inhibitor dasatinib may actually induce pulmonary hypertension [5,6,7]; it was more recently suggested that this dasatinib effect may in fact be independent of the src inhibition [7]. This family of kinases has been recently shown to be involved in the subgenomic RNA translation and replication of alpha-viruses, their inhibition being put forward as a potentially effective way of treating infections with such viral particles [8]. A constant interest for understanding the pharmacology of this class of compounds, as well as for developing new src inhibitors, may open the doors wide for multiple therapeutic applications for these inhibitors in a variety of pathologies.

The first member of this family (c-src) may play a more significant role than other members of the same family in certain pathologies or clinical contexts. For instance, c-src, but not Lyn and Fyn src kinases, is upregulated by hypoxia and has an important part in prostate cancer metastasis of hypoxic tumors (hypoxia is a negative prognostic factor in this malignancy) [9]. Furthermore, c-src tyrosin kinase has been shown to be abnormally activated or overexpressed in a number of different malignancies and to stimulate processes associated with tumor progression, such as proliferation, angiogenesis, or metastasis [10]. Src tyrosin kinase inhibitors have been explored as potential new therapeutic agents in a variety of malignancies such as melanoma (one such inhibitor demonstrating in vitro activity on a variety of melanoma cells, including some BRAF^V600^ mutant cells [11], but a report that src inhibition would induce melanogenesis in melanoma cells has also been published [12]), papillary thyroid carcinoma [13], clear-cell renal carcinoma [14], pancreatic [15], or ovarian cancer [16].

The space of the universe is expanding, but so is the “chemical space”. Currently PubChem includes some 96 million different chemical compounds [17], an impressive number, but minuscule when compared with the number of chemical compounds that might be synthesized in the coming years. GDB-17, probably the largest database of molecules up to date, included in 2015 no less than 166 billion compounds, and these are limited to only a few types of atoms (C, N, O, S, and halogens) and a maximum of 17 atoms per molecule [18]. Theoretical calculations using constraints for circumscribing the drug-like chemical space have suggested that the number of molecules obeying to the Lipinsky’s rules is about 10^33^ [19], an estimate intermediary between 10^60^ (as proposed earlier by R.S. Bohacek et al. [20]) and 10^23^ (as advanced later by P. Ertl. [21]). This raises questions regarding how to assess all these substances for their pharmacological, toxicological, or biological effects (in all contexts, for all targets etc.). While it is simply “mission: impossible” by the traditional route of wet lab experiments, the relatively cheap computing power available today may offer surprisingly good results (although far from perfect).

Built on three pillars (biological data, chemical knowledge, and modeling algorithms), QSAR (quantitative structure-activity relationship) [22] methodologies allow the development of computational tools for predicting with reasonable confidence (when validated appropriately) a wide variety of biological activities from the molecular structure of chemical compounds. Although the QSAR approaches have not gained in popularity as fast as the molecular docking modeling, the field has been far from being inert in the last decade or so, with various new approaches to the mathematical algorithms used or the biological activities explored [23]. The models developed and validated may then be applied for virtual screening of a large number of substances, allowing the quick identification of a sizeable number of compounds of interest (with certain activities or biological properties). Such virtual screening exercises may be further coupled with other computational methods, such as ligand-target docking for confirmation of activity [24,25]. Whereas the classical drug development process is very costly and tedious, computational methods have a high efficiency and are inexpensive [26]. In this context, we developed a set of QSAR models with different descriptors and machine learning classification algorithms, integrated by stacking, to be used for virtual screening of c-src tyrosin kinase inhibitors. A number of 49 QSAR models with reasonably good performance were selected and applied for the virtual screening of over 100,000 chemical compounds from the ZINC database [27]. A total of 147 compounds with the highest probability of being active were also assessed by molecular docking resulting in 89 compounds where the docking data were consistent with a hypothesis of activity. Data from ChEMBL and PubChem externally validated the virtual screening results for a number of compounds.

## 2. Results

### 2.1. Dataset Analysis

**Figure 1:**
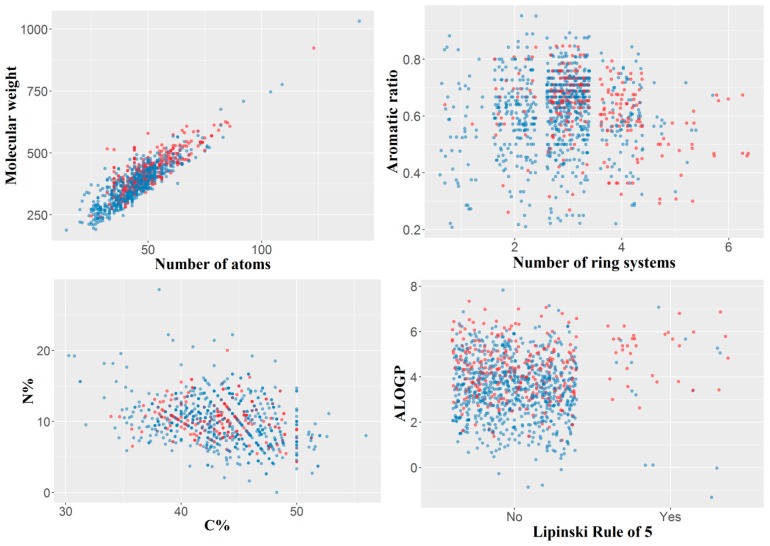
Variability of the dataset illustrated by several simple constitutional descriptors or molecular properties. Blue—inactive compounds; red—active compounds. For the Lipinsky rule, “No” indicates compounds not obeying to the Lipinsky’s rule of five, and “Yes” compounds satisfying the rule; among the latter the active compounds are more frequent. C% indicates the percentage of carbon atoms, N% the percentage of nitrogen atoms, whereas ALOGP is the Ghose-Crippen octanol-water partition coeff. (logP).

In our study, the final dataset included 1038 small organic molecules with a molecular weight varying from 188 to 1032 Da, a range usual in the QSAR modeling, with a median value of 390 Da and 75% of the values smaller than 440 Da. The number of atoms per molecule varied between 14 and 143, the median and mean values being 46 and 46.6, respectively. All molecules had at least one ring system and a maximum of six rings (with a median of three). Only 46 of the 1038 molecules satisfied the Lipinsky’s rule of five, of which 32 were labeled as “active” (ki < 1000 nM), and 14 as “inactive” (ki ≥ 1000 nM). The variability of the dataset by several simple constitutional descriptors or molecular properties is presented in Figure 1.

**Figure 2:**
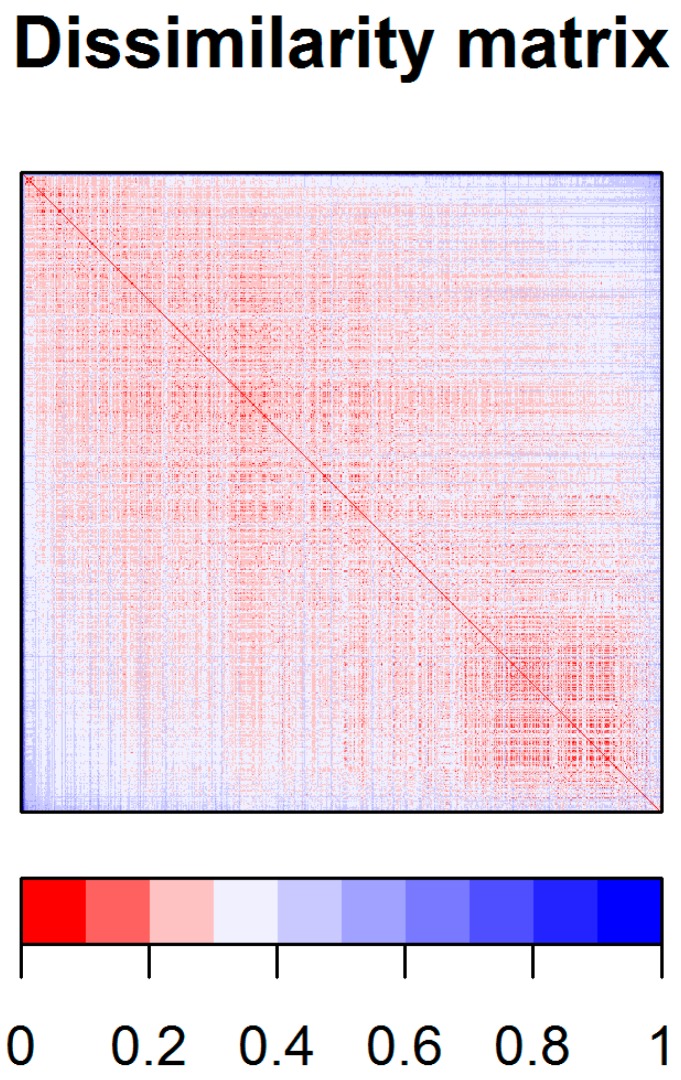
Dissimilarity matrix illustrating the variability among the dataset based on the Gower distances between the compounds.

A dissimilarity matrix based on the Gower distance was computed (the Gower distance is appropriate for data of a heterogeneous nature), using 783 most relevant descriptors (that remained after removing autocorrelated and quasi-constant features). Although Gower distance takes values between 0 and 1, because it tends to give larger weights to binary variables [28], we rescaled the distance matrix and plotted it as a dissimilarity plot (Figure 2). Before rescaling the maximum value of the Gower distance was 0.404, following rescaling it became 1. The median (scaled) dissimilarity values were mostly around 0.2–0.3, suggesting that the chemical diversity in the dataset was rather limited (Supplementary Figures S1–S3).

### 2.2. Performances of Models in Nested Cross-Validation

Using a variety of classification algorithms (random forests, support vector machines, adaboost M1, Bayesian Additive Regression Trees, C5.0, and binomial regression), of feature selection methods (17), and numbers of features (between 3 and 40—for instance, for binomial regression we used models with 3, 5, 10, and 20 features, and thus the number of models built for this classifier was 68), a total number of over 350 models were built and their performance was assessed by nested cross-validation. Only models with an acceptable performance (defined as having both a balanced accuracy higher than 70% and a positive predictive value higher than 70% in the nested cross-validation) were selected (Table 1). In instances when several models (with different numbers of features) had good performance for the same classifier and selection algorithm (over the threshold of 70%), we only tabulated the model we judged as best (highest average between balanced accuracy and positive predictive value (PPV), and for equal value of the average giving preference to higher PPV). Numbers of true positives, true negatives, false positives, and false negatives, allowing computation of other performance metrics are available in Supplementary Table S2.

As the dataset includes 1038 compounds, of which 286 are active and 752 inactive, the most probable random accuracy (Q_2,_ rnd) [29,30] may be estimated to 60.08% (286 × 286 + 752 × 752)/(1038 × 1038). As shown by the last column in Table 1, our models have a superiority of about 20–24% over random accuracy. However, the concept of random accuracy assumes that the correct classification of the two classes is of equal importance; in fact, in our case, we were more interested in correctly predicting the active compounds (i.e., optimizing the PPV was more important than Q_2_). The models were thus not optimized to increase the global accuracy, but rather both balanced accuracy and PPV.

As the models applied in the nested cross-validation are always based on only a subset of the data, the estimation of performance should be conservative (i.e., applying the selected models on the whole dataset has better performance).

### 2.3. Y-Randomization Test

As expected, despite following the same steps in building the models, scrambling the activity labels had a strong impact on the performance of the models, which was clearly inferior to those based on the initial (unscrambled) data: the average balanced accuracy of all 10 y-scrambling tests (nested cross-validation performed in the same conditions and following the same pre-processing as the true data) was 50.23%, with a standard deviation of 0.59% (minimum value 49.73% and maximum 51.45%). In a similar way, the mean value of the positive predictive (PPV) was 20.38%, and its value varied between 0.00% and 30.00%. This provides reassurance that the performance of the models is not the result of mere chance, but rather reflects a true relationship between the descriptors and the inhibitory activity on c-src tyrosine kinase.

### 2.4. Descriptors Associated with c-src Inhibitory Activity

While for all models the number of features was relatively high (in most cases between 20 and 30), the largest predictive effect could be attributed to no more than five features. For instance, in the case of random forest, using ANOVA as a feature selection (filtering) algorithm, with 23 features, the area under the receiver operating characteristic (ROC) curve (AUC) was 82.56% and the balanced accuracy 70.24%; however, using only the first most important five molecular descriptors, the AUC was 77.53%, and the balanced accuracy 66.39%. Although there was an improvement for the higher number of features (23), the first five explained the largest part of the variability in the training and testing datasets. We therefore focused on the first five descriptors selected by each of the 17 selection algorithms and found that most algorithms identified the same features as being the most important. These are shown in Table 2 (and descriptor values in Supplementary Table S3).

### 2.5. Virtual Screening and External Validation

We applied the models to the 104,619 ZINC compounds and ranked them based on the percentage of models predicting the compounds as active. Using a threshold of 50% (i.e., compounds predicted to be “active“ by more than 50% of all models applied) 744 compounds were identified. Our validation data (using the predictions on the test sets from the nested cross-validation) indicated that the PPV for this threshold was 78.57%. Increasing the decision threshold to 75% the number of compounds decreased to 158, but after eliminating the compounds that had been part of the training set and the duplicates (multiple ZINC ids may correspond to the same substance), their number decreased to 115 (Table S2); the validation data indicated a PPV value for this threshold of 85.43%. For a threshold of 90% the PPV in the validation was also close to 90% (90.1%), but the number of unique compounds was limited to 37.

For external validation purposes, we searched PubChem and ChEMBL for biological data related to the activity of the predicted compounds on the src tyrosine kinase, so as to have at least partial confirmation on the accuracy of the predictions. We found that among the 115 substances predicted as being active, for nine compounds (i.e., 7.83%) there is available evidence that they are active on the c-src tyrosine kinase. We could not find ki values for the nine compounds, but in most cases rather mean inhibition (as a percentage) at 1.0 or 0.1 μM was available. Taking into account that IC50 values are always higher than ki values for a competitive inhibitor, and the fact that percent inhibition is dependent on both substrate and inhibitor concentration, we considered as active compounds those with inhibition values of at least 30%. When a compound was labeled as “active” on the src target in one of the two public databases without further information on the endpoint or bioassay used, we also considered that compound as active (that was the case for balamapimod, reported by PubChem). Of the nine compounds labeled by us as “active”, three had a mean inhibition higher than 50%, one had a ki less than 1000 nM (20 nM to be precise), one was stated as “active” by PubChem with no further information and four had a mean% inhibition between 30% and 42.23% at 1 μM). A total of 34 additional substances (29.56%), predicted by the large majority of models as being active, were in fact proven to be inactive on src-tyrosine kinase, whereas 72 of the substances (62.61%) predicted to be active, seem to have never been tested for their effect on src tyrosine kinase. If the 43 compounds that were indeed tested were representative for the rest, the rate of success for the predictions would be 20.93%.

### 2.6. Applicability Domain

The “applicability domain” (AD) is a concept meant to evaluate if a model may be validly applied to predict the effect of a candidate compound; such validity is conditioned on the satisfaction of the assumptions applied in the construction of the model [31]. If the new substance whose activity we are trying to predict differs substantially from those on which a QSAR a model was based, such a prediction cannot be trusted. Therefore, assessing the AD for model is of paramount importance if that model is to be used for predictions, and a wide range of methods have been proposed in the literature for this purpose, each with its own advantages and flaws [32].

**Figure 3:**
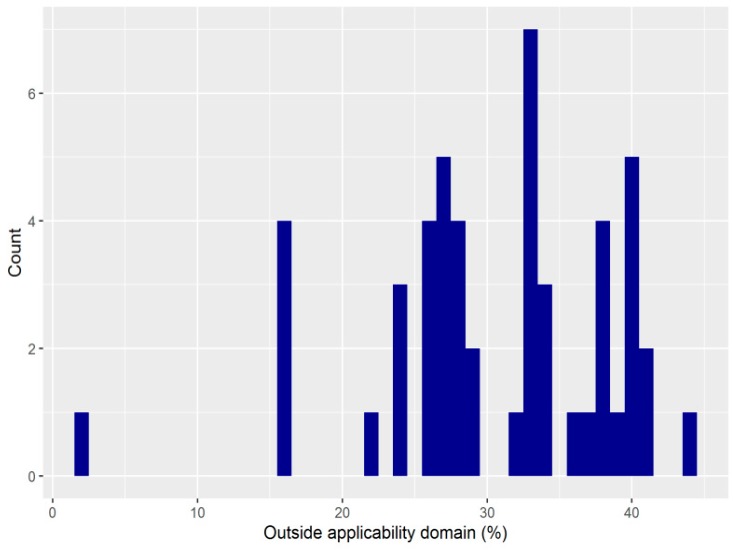
Variation of the proportion of compounds estimated to be outside the applicability domain (F. Sahigara et al. method [34]) for the 49 QSAR models used in virtual screening.

We used a variety of algorithms to assess the applicability domain for the predictions of the QSAR virtual screening by different models. Using the method by Roy et al. (2015) [33], which considers as an outlier each compound with a value outside the mean ± standard deviation, none of the compounds predicted by more than 50% of our models to be active were outside of the applicability model. This was not very surprising, because that method uses a decision tree based on three standard deviations, whereas we capped, centered, and scaled values to two standard deviations. Using the Kernel Density Estimation Outlier Score (KDEOS) algorithm (with a minimum of three and a maximum of 10 neighbors), which is based on a number of k-nearest neighbors, the number of outliers among the 744 compounds predicted as active by the majority of the QSAR models was small for each model, and not higher than 15% of the total number (with a median proportion toward 5%). Selecting the compounds after filtering them based on the applicability model did not change the hierarchization of the compounds predicted as active. The Influenced Outlierness (INFLO) algorithm (with k = 5), which is also based on a number of k-nearest neighbors, but taking into account a “a reverse nearest neighborhood set”, and that of F. Sahigara et al. (2013), which not only uses k-nearest neighbors, but also individual decision threshold for each data point of the training sample [34], identified a much larger proportion of compounds as outside the applicability method: for the latter, for instance, the proportion of outliers varied (for the different models) between 1.75% and 44.35%, with a median of 32.39% of the total of 744 compounds (Figure 3). A number of 147 compounds (of which five had been in the training dataset) were predicted by 75% of the models as being active, after limiting the votes to those compounds that were within the applicability domain estimated with the F. Sahigara et al. (2013) method [34]. All compounds identified by the virtual screening (before checking the applicability domain) fell for at least some of the models within the applicability domain, but the degree of confidence in the predictions changed after checking for the applicability domain.

### 2.7. Molecular Docking

**Figure 4:**
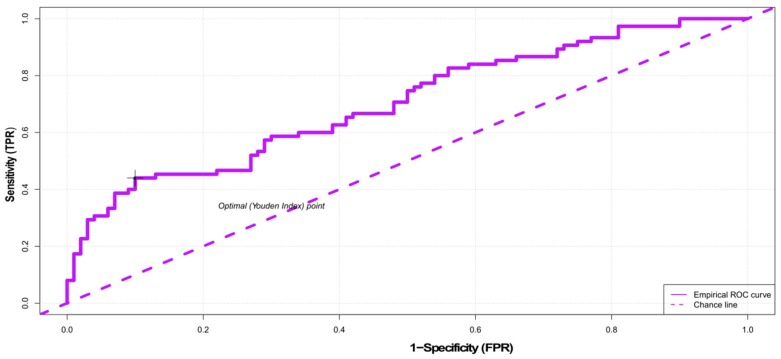
Receiver operating characteristic curve for the performance of molecular docking using LeDock software on the training set (*n* = 175 compounds, as described in the text).

**Figure 5:**
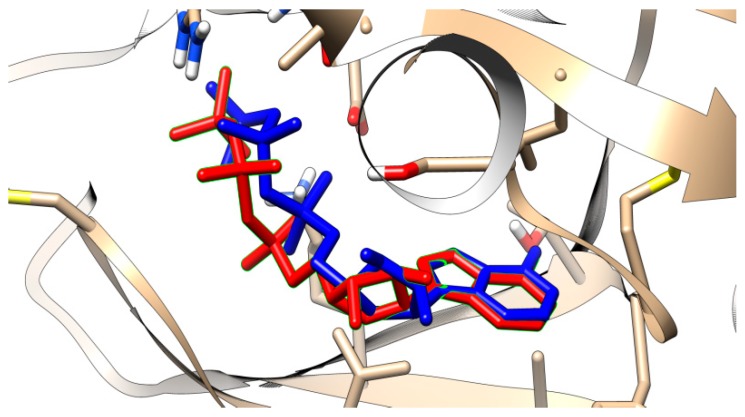
Crystallographic pose of the NAP ligand within c-src tyrosine kinase (in red) and predicted pose by LeDock (in blue). It may be seen that the rings overlap very closely, whereas the free aliphatic chains do not overlap so well.

In order to assess the performance of docking for the two software programs used (AutoDock Vina [35] and LeDock [36]) we first compared the estimated energies of binding for 175 compounds of the training set, with known activities on the target enzyme. With LeDock, the mean binding energy in the active compound group was −8.02 kcal/mol, whereas in the inactive compound group it was −7.29 kcal/mol (*p* < 10^−7^, Welch t-test). For the very active compounds (ki < 20 nM), the mean binding energy was −8.43 kcal/mol (*p* < 10^−8^ versus all inactive compounds, Welch t-test). Using the “cutpointr” package, an optimal cut-off was found at an energy of binding of −7.17 kcal/mol, which ensured an accuracy of 70.29%, with high sensitivity (90%), but low specificity (44%). In order to minimize the false positive, a cut-off point of −9.21 kcal/mol was necessary; at this level the specificity was 100% (i.e., none of the inactive compounds had such a low energy of binding in the docking runs), but with a very low sensitivity (only 9% of the active compounds had this low estimated energy of binding) (Figure 4). As our interest was to minimize the false-positive rate, we docked the 147 compounds predicted by the QSAR models to be active and within the applicability domain and somewhat surprisingly no less than 89 of them (61.22%) had such a low energy of binding, in other words they could be considered as active (Table 3). Considering that in our training subset, the sensitivity at this cut-off point (−9.21 kcal/mol) was only 9%, this high value does suggest that an important proportion of the compounds predicted by the QSAR models to be active might be indeed active, although when using docking one must be very cautious [37]. The root-mean-square deviation (RMSD) computed for the first cluster of poses of the ANP was 1.25, under the conventional threshold of 2.0, which may be considered reasonably well. The visual examination of the pose indicated that the ring pose was very well predicted, whereas the side chain prediction was less accurate (Figure 5). Of the 89 compounds of Table 3, 34 (38.20%) have already been reported to inhibit one or multiple tyrosine kinases.

Following the suggestion of one of the reviewers of this paper, we also submitted the 89 compounds to the online version of PASS [38], a software that predicts potential activities for chemical compounds. A total of 24 out of the 89 compounds (26.97%) were predicted to be active on the src tyrosine kinase and 62 of the 89 compounds were predicted to be active on at least one or multiple kinases (Table S4, Supplementary Information). Nevertheless, PASS predictions are also affected by limitations, because Pf-562271, a compound that was in our training set, was not detected at all as a src-tyrosine kinase inhibitor. Gw683134a, which based on the ChEMBL data causes a 36.99% inhibition of c-src tyrosine kinase at 1 μM, was not predicted as an inhibitor at all. Bx-795, which also at 1 μM causes a 27–30% inhibition of human c-src and 77–90% inhibition of *Gallus gallus* c-src, was also not predicted as an inhibitor. As for lapatinib, the probabilities to be active and to be inactive predicted by PASS were only 0.086 and 0.053, respectively.

AutoDock Vina performance was inferior to that of LeDock: on the same 175 compounds from the training set, the mean energy of binding was −10.30 kcal/mol for the active compounds and −10.03 kcal/mol for the inactive (*p* = 0.21, Welch t-test). An optimal cut-off for the AutoDock Vina compounds was at −9.26 kcal/mol, which ensured an accuracy of only 62.86%, with a sensitivity of 87.00% and a specificity of only 30.67%. As the performance of Vina was inferior to that of LeDock, we preferred to use only LeDock for virtual screening.

Computing various ligand efficiency metrics did not improve the predictions in the case of LeDock results: the accuracy rather decreased with all ligand efficiency measures attempted. In the case of AutoDock Vina, using different ligand efficiency measures changed the values of accuracy, sensitivity, and specificity, with no spectacular improvement. For instance, dividing the energy of binding to the molecular weight decreased sensitivity (from 87% to 43%), increased specificity (from 30.67% to 81.33%), and slightly increased the AUC (from 56.85% to 62.87%), but it also slightly decreased the accuracy (from 62.86% to 59.43%). Of the different ligand efficiency measures, for the AutoDock Vina results the best was obtained by dividing the energy of binding to the squared Ghose–Crippen octanol-water partition coefficient: 78% sensitivity, 49.33% specificity, 65.71% accuracy, and 65.05% AUC. Even with this ligand efficiency measure, the results were inferior to those obtained with LeDock based on the energies of binding.

## 3. Discussion

Several studies of QSAR models for c-src tyrosine kinase inhibitors have been published up to date in the scientific literature. Five such studies have explored the use of 3D-QSAR, and all of them used a relatively small number of compounds (80, 42, 156, and 39, respectively), with the same basic chemical structure within each study (pyrrolo-pyrimidine, quinazoline, anilinoquinazoline and quinolinecarbonitrile, quinolinecarbonitrile, and 4,6-substituted-(diaphenylamino)quinazolines); they could, therefore, be considered “local” models [39,40,41,42]. In the QSAR field, the term “local” is used to designate models based on a data set consisting of compounds related by their chemical structure, unlike global models, that are based on data sets consisting of structurally diverse chemical substances [43]. Another paper reported on the use of 2D-QSAR for c-src inhibitors, but these models were also local, focused on ethynyl-3-quinolinecarbonitriles [44]. Therefore, our study is the first one focused on global QSAR models for inhibitors targeting the c-src tyrosine kinase. It has been argued (and it stands to reason) that local models tend to have limited predictive power, even when their apparent performance indicates that they are robust [43]. Our global models are expected to have a higher predictive power, as partially confirmed in our external validation.

By far the most important descriptor in our work, identified by multiple feature selection algorithms, was SpMax4_Bh(m), the largest eigenvalue n. 4 of Burden matrix weighted by mass. This has not generally been reported in previous works as correlating with pharmacological activities. Other two Burden eigenvalues (SpMax3_Bh(m), SpMax5_Bh(m)) have also been among the most important descriptors correlating with the inhibition of c-src. SpMax3_Bh(m) has been used in predicting depuration rate constants for environmental pollutants of the polychlorinated biphenyls group [45], and the less relevant (in our case) SpMax6_Bh(m) has been used to predict chronic toxicity of substances to *Pseudokirchneriella subcapitata* [46]. The second most important descriptor for our data set was DECC (eccentric topologic index), which has been previously reported to be important in the prediction of monoamine oxidase A (MAO-A) activity [47,48], placental barrier permeability [49], and gas chromatographic retention times [50]. F06[C-N] was used in a model to describe the anti-proliferative effect of phenyl 4-(2-oxoimidazolidin-1-yl)-benzenesulfonates (local QSAR model) [51], antimalaric effect [52], or skin permeability of substances [53]. P_VSA_MR_6 has also been used for modeling of skin permeability [53], whereas we identified the use of Chi1_EA(dm) only for the QSPR modeling of fluorescence properties of a number of fluorescent dyes [54]. The aromatic nitrogen (N-073) has been shown to correlate positively with HIV-1 integrase activity inhibition [55] and negatively with the inhibition of the fibroblast growth factor (FGFR) [56]. We found no previous reports on the use of the Balaban distance connectivity index (J_D) in other models in the biological field, neither of the F05[C-N].

Rarely, the 49 QSAR models with similarly good performance converged in their predictions. Only eight compounds were predicted by all models to be active, and half of them (*n* = 4) were already in the training data set; for the large majority of compounds at least one or more of the models had contradictory results. This illustrates the need to avoid making decisions based on the results of a single or a small number of models.

As shown in the results section, for nine compounds (7.83% of the 115 substances with the best predictions) it has been confirmed that they are active. How good is such a measure for a virtual screening exercise? If we compare it with the PPV value in the nested cross-validation, the results are rather disappointing and indicate that one should always be cautious in interpreting results even when using double cross-validation, because the real world data are likely to be different from the data set used for training and testing. For instance, it is likely that the proportion of actives in the available data set used for the construction of the models is higher than the proportion of actives in the “real world“ (i.e., the wide chemical space used for virtual screening), and this may lead to a decrease in the positive predictive value in the real world. However, if we compare the results of the virtual screening with those of the most costly high throughput screening (HTS), the results are noteworthy. It has been reported that the hit rate of HTS should be expected to be less than 1% [57] and even less than 0.1% or 0.01% [58]. In one study, adding a computer-aided virtual screen was able to increase the screening hit proportion to 5.8% [57]. Thus, our success rate of at least 7.83% is reasonably good. If we compute the confirmation rate against the compounds that were assessed for their effect on src-tyrosine kinase (20.93%), the results are even better. As another positive aspect, more than a quarter of our predictions were supported by the PASS online software. Our virtual screening results showed, however, additional interesting facts.

A total of 16 additional false positives were in fact reported to be active on other members of the src family members, particularly Yes1 tyrosine kinase. This suggests that although our virtual screening exercise failed in multiple cases, the failure was often not far from the true target. Thus, from a total of 43 molecules that were tested for their effects on the src and other tyrosine kinases, 58.14% (25 compounds) were inhibitors of one or several members of the src-tyrosine kinase family (most often Yes1, sometimes also LCK or LYN tyrosin kinase).

Other false positives of the virtual screening exercise are inhibitors of proteins that src tyrosine kinase interacts directly, either activating them or being activated by them. It is known, for instance, that EGFR (epidermal growth factor receptor) can be activated by src without the presence of the EGFR ligand and that there is a direct correlation between EGFR overexpression and src activation [59]. Rather surprisingly for us, 13 compounds wrongly predicted by our models to be src tyrosine kinase inhibitors, are in fact inhibitors of EGFR, and 10 additional compounds that were inactive on src or other members of src family, were reported to be inhibitors of EGFR. Most of these 10 additional compounds (as well as most of the compounds active on src or Yes1 tyrosine kinase) are also active on ErbB4, and it has been reported that ErbB4-derived phosphopeptides are able to interact with the SH2 domain of src [60], that following stimulation by EGF, c-src is rapidly recruited to ErbB receptor complexes [61] and that activated src binds to ERBB4s80 (E4ICD), a cleaved fragment of ERBB4 [62]. Moreover, dasatinib, described often as a src inhibitor [63], has also shown to be one of the most potent ligands of ErbB4 [64]. Defactinib, apparently a false positive of our virtual screening is a potent FAK (focal adhesion kinase) inhibitor; it is known that FAK and nonreceptor src tyrosin kinase are both part of a focal adhesion complex (together with other structural, enzymatic, or adapter proteins), where they interact directly [65]. Three false positives of the virtual screening results were KIT and PDGFR inhibitors; KIT promotes phosphorylation of src and is activated by src [66], while src and PDGFR interact and phosphorylate each other at certain Tyr positions [67].

Such findings (compounds inactive on c-src tyrosine kinase, but active on kinases from the same kinase family or signaling pathway) tend to suggest that where the QSAR virtual screening fails is often not far from the target (but this is nonetheless a failure). How could these failures be explained, considering that multiple models converge in predicting a certain molecule as active on the target of interest (src tyrosine kinase)? It seems that the models manage to predict the tyrosine kinase properties of certain compounds, without having sufficient specificity to always separate those active on src from those active on other tyrosine kinases. We hypothesize that the training set is too small and does not include (a sufficient number of) molecules with selective src inhibitory properties; we intend to evaluate whether extending the data set with additional molecules inactive on src but active on other tyrosine kinases may improve the results of the virtual screening. It is also worth exploring the combining of more diverse descriptor sets in the final assembly of models with a view of improving the performance of the virtual screening.

Among the results produced by our virtual screening there is a sizeable number of antiviral molecules (vedroprevir, daclatasvir, ciluprevir, deleobuvir, ledipasvir, faldaprevir, tegobuvir, elbasvir, ombitasvir, narlaprevir), all of them approved or developed against hepatitis C viruses. They either target the NS3/NS4A (vedroprevir, ciluprevir, faldaprevir, narlaprevir) [68] or NS5A (daclatasvir, elbasvir, ombitasvir, ledipasvir) [69] or NS5B (deleobuvir, tegobuvir) [70] nonstructural proteins of the virus. It is not very surprising to see inhibitors of NS5A and NS5B here, considering that is already known that NS5A protein binds to tyrosine kinases from the src-family [71], and c-src is an essential host protein involved in the formation of the HCV replication complex, together with NS5A and NS5B [72]. It was less expected to see also inhibitors of the NS3/NS4A among the results of the virtual screening, because no direct interaction was reported between the Ns3/NS4A complex and src tyrosine kinase. This list of HCV antivirals might consist only of false positives, but it is worth testing in wet lab experiments.

The docking applied to 147 compounds predicted with a high probability by the QSAR models to be active, reduced their number to about 61% of the initial size. For a number (27.78%) of these 89 compounds, predicted by both QSAR and docking to be active, data available in ChEMBL or PubChem (from a single wet lab test) indicate that they are inactive, and for others (6.67%), that they are active, as discussed for the QSAR models. This suggests that computational results have to be interpreted with caution even when different models, with different methodologies and assumptions, converge in their predictions. On the other hand, the last decade has witnessed a growing realization of what has been dubbed “the reproducibility crisis”, ascribed to the inappropriate quality of antibodies used as reagents [73], insufficiently described methodologies or simply to the biology itself [74]. Whereas positive findings have often not been reproduced when experiments were repeated in other laboratories, it is not impossible that negative findings could also not be replicable and some of the compounds shown by databases to be inactive might, as a matter of fact, be active. However, in the absence of contrary evidence, such compounds have to be considered inactive.

Virtual screening results are also influenced by potential errors affecting the input data: if the wet lab data that were used to generate the models are affected by errors, they will propagate forward in the models built and in the predictions made on new compounds. The estimated docking energies are also potentially affected by errors (in our estimation the accuracy was about 70%, but the large number of compounds used in screening may differ more from our data set, and thus accuracy might be lower). Moreover, docking methods are also prone to errors, there are often discrepancies between docking results and ligand-based studies, and there are multiple cases where top compounds identified by docking methods failed in wet lab experiments [37].

## 4. Materials and Methods

### 4.1. Dataset

The dataset (Table S1) was downloaded from ChEMBL (https://www.ebi.ac.uk/chembl) and included experimental data for c-src as a target (target code CHEMBL267). Only the records with ki values expressed in nM were kept. Records with “=” values in the field “Relation” were kept for analysis and labeled as “active” if ki < 1000 nM and “inactive” if ki ≥ 1000 nM; records with “>” or “<” values in the field “Relation” were kept for analysis only if they allowed unequivocal classification (e.g., records with ki > 5000 nM were kept and labeled as “inactive”, whereas those with ki > 100 nM were discarded; similarly, records with ki < 5000 nM were discarded). A threshold of 1000 nM for the formal discrimination between “active” and “inactive” compounds is usual in the field and has been used in other publications [75]. We used classification rather than regression, because the data came from different laboratories and experimental settings, and although ki values have less variability than IC50, published experimental ki values still vary considerably (of the 75 compounds in our data set with multiple ki values, the relative standard deviation (RSD) of ki varied from 0% to 103%; for the first three quartiles, RSD was relatively low, under 13.85%, but for the last quartile it was quite high). Inorganic compounds were removed. For the detection and removal of duplicate compounds we proceeded in two steps: first, canonical SMILES (available in the downloaded dataset) were searched for duplicates in R (v. 3.6.0) and their ki values were replaced by the average of the duplicates. We then used ChemAxon Standardizer v. 18.8.0 (ChemAxon, Budapest, Hungary) for the standardization of the molecules, and then employed the ISIDA/Duplicates software (http://infochim.u-strasbg.fr; University of Strasbourg, Strassbourg, France) software for the identification of potential further duplicates. We used Discovery Studio Visualizer v16.1.0.15350 (Dassault Systèmes BIOVIA, San Diego, CA, USA) to convert the standardized SMILES to 2D chemical structures (sdf). Following the removal of duplication, our dataset decreased from an initial number of 1151 compounds to 1038, of which 286 were labeled as “active” and 752 as “inactive”.

### 4.2. Descriptors

Molecular descriptors of the dataset molecules were computed using the Dragon 7 software (version 7.0, https://chm.kode-solutions.net; Kode SRL, Milano, Italy). A total of 19 blocks of molecular descriptors were computed: constitutional descriptors (*n* = 47), ring descriptors (*n* = 32), topological indices (*n* = 75), walk and path counts (*n* = 46), connectivity indices (*n* = 37), information indices (*n* = 50), 2D matrix-based descriptors (*n* = 607), 2D-autocorrelations (*n* = 213), Burden eigenvalues (*n* = 96), P-VSA-like descriptors (*n* = 55), ETA indices (*n* = 23), edge adjacency indices (*n* = 324), functional groups count (153), atom-centered fragments (*n* = 115), atom-type E-state indices (*n* = 172), CATS 2D (*n* = 150), 2D atom pairs (*n* = 1596), molecular properties (*n* = 20), and drug-like indices (*n* = 28). All descriptors thus computed were 3839.

### 4.3. Feature Selection

As the number of computed descriptors is very large (almost 4000), the “dimensionality curse” precludes optimal operation of the classification or regression algorithms, which are generally designed for a relatively small number of variables, and tends to result in overfitting [76]. Feature selection, which is a process of filtering a high number of variables while keeping only the most relevant of them increases the performance of machine learning algorithms, reduces the computational costs, and strengthens the generalization ability of the models built [76]. Multiple algorithms of feature selection have been proposed in the literature, with variable performance, often depending on the nature and particularities of the data. We used 17 different feature selection algorithms, implemented directly in the “mlr” R package [77] or through other R packages: based on an ANOVA test, on a Kruskal test, on the Area Under the Curve (AUC), variance, and an univariate model performance score (‘mlr’), based on a permutation importance of random forest (as implemented in the R package ‘party’, [78]), based on a chi-square test, gain ratio, information gain, OneR classifier, RELIEF algorithm, and symmetrical uncertainty (methods implemented in the ‘FSelector’ R package [79]), three algorithms based on random forest importance (as implemented in the randomForest [80] and randomForestSRC [81] packages), and two algorithms based on node impurity and permutation in random forests, as implemented in the ‘ranger’ R package [82]. The feature selection algorithms were applied after pre-processing consisting of removal of constant and quasi-constant features (i.e., those where less than 1% of the observations differed from the mode value) and highly correlated features (defined as those with a correlation coefficient higher than 0.9).

### 4.4. Machine Learning Algorithms and Model Building

For building the models we used the following algorithms: random forests, support vector machines, ada Boosting M1, Bayesian additive regression trees, binomial regression, and C5.0 decision trees and rule-based models.

Based on an arbitrary number of decision trees used as an ensemble with a majority vote to decide on the most probable class assigned to each data point, random forests (RF) are a popular classification algorithm often used with very good performance in QSAR models [83,84,85]. Each decision tree is constructed using bootstrap sets of the training set and subsets of descriptors that are selected in a random manner [86].

The support vector machines (SVM) algorithm is able to address data sets with high number of variables and has often been used with very good performance in a variety of classification and regression tasks, including QSAR applications [87,88]. It uses a variety of kernel functions (e.g., linear, polynomial, radial, etc.) to project features in a vector space maximizing the partitioning boundary between classes and to identify the hyperplane that best discriminates the classes [89].

The adaboost M1 (Adaptive Boosting) algorithms were described as “widely used in QSAR studies” [90], although they are probably less used than RF or SVM. AdaBoost is an iterative algorithm that uses weights to improve the performance of “weak” classifiers (particularly decision tress), giving higher weights to the trees with better performance (smaller misclassification rates) [90].

Bayesian Additive Regression Trees (BART) is nonlinear regression technique based on a Bayesian approach, whose performance in QSAR modelling has been stated to be competitive with that of other machine learning methods [91]. Unlike other decision trees, where decision is taken based on a majority vote or with the help of empirical weights, BART makes use of prior knowledge and likelihood to improve the performance of the decision trees.

Binomial regression (logistic regression), despite the term “regression” is a relatively simple algorithm used for classification purposes, because it linearly models the probability that an observation belongs to one of two categorical outcomes [92]. In other words, logistic regression computes the probability P = 1/(1 + e^−t^), where t = a_0_ + a_1_x_1_ + a_2_x_2_ + ... + a_n_x_n_ [93].

C5.0 decision trees and rule-based models represent an extension of a classification algorithm proposed by R. Quinlan in 1993, under the name “C4.5”, and builds models that can take either the form of a decision tree or a set of rules (in simple or boosted versions) [94]. Although apparently less used in QSAR modeling than other machine learning algorithms, when employed, it gave excellent performance, comparable with that of random forests or support vector machines [95].

All models were built and their performance was assessed in the computing and programming environment R, v. 3.6.0 [96], using ‘mlr’ package [77] coupled with “parallelMap” [97] for parallel computing, and to a small extent, the “caret” package [98]. Classification algorithms were used from the corresponding R packages implementing them: ‘randomForest’ [80], ‘e1071′ [99] (for SVM), ‘RWeka’ [100,101] (for adaboost M1), ‘bartMachine’ [102] (for BART), ‘stats’ [96] (for the logistic regression), and ‘C50′ (for the C5.0 algorithm) [94]. Gower distances were computed with the “cluster” R package [103]. Graphs were built in “ggplot2” [104] and (for the dissimilarity plot) “seriation” [105]. All values were standardized by centering and scaling, and values larger than two standard deviations were capped to 2.

### 4.5. Performance Evaluation

Nested cross-validation using five folds in the inner loop and 10 folds in the outer loop was used to evaluate the performance of the models selected, except for the Bayesian Additive Regression Trees, for which five folds were also used in the external loop (due to the long time taken by this classifier). The assessment of QSAR model performance should include both internal and external evaluations, and the external validation is generally deemed as “the gold standard” [106,107]. However, the concept of “external validation” has received different interpretations and most often is assumed to describe a holdout data set, obtained by an initial one-time split (i.e., a set that has not been seen by the model during any adjustments or hyperparameter optimization) [108]. Despite its apparent advantages of objectivity and ability to assess the generalization of the selected model(s), the use of a hold-out data set is fraught with thorny issues: the split may be simply fortunate, leading to overestimation of performance (or of contrary, it may be unfortunate, leading to underestimation of performance), it requires the holdout sample to be large (which in practice may be costly or a requirement impossible to satisfy), and the sample size needed for holdout is larger than it is necessary for cross-validation to estimate the prediction error with a similar degree of precision [106]. For these reasons, using nested cross-validation (also known as double cross-validation) not only does not reject the idea of external validation, but it extends it to the entire data set [109].

All models were assessed by computing (within the nested cross-validation) the balanced accuracy (BA), mean misclassification error (MMCE), sensitivity (true positive rate, TPR), specificity (true negative rate, TNR), area under the receiver operating characteristics curve (AUC), and positive predictive value (PPV), with their widely known definitions and equations [75,110] (formulae for their computation are available in the Supplementary Information). Particularly for virtual screening purposes PPV is important (because it indicates the likely proportion of positive values among the values predicted as positive). We therefore selected only models with a PPV higher than 70% and BA higher than 70%.

To make sure that the performance of the models is not consequential to chance, a Y-scrambling procedure was applied, where for multiple models the dependent variable (in our case the ki values) was shuffled through 1000 permutations (using the R package ‘gtools’ [111]), then the models were rebuilt using the same procedure from the first steps (i.e., applying the same feature selection algorithms, in the same order) and their performance evaluated. If there is a real relationship between the activity and the descriptors, following the y randomization the performance of the new models thus built should be worse.

### 4.6. Applicability Domain

We used two local density-based outlier methods implemented in the DDoutlier R package [112]—the Kernel Density Estimation Outlier Score (KDEOS) algorithm with gaussian kernel [113], and the INFLO algorithm (which compares the density in the neighborhood of an observed value with the density in the “reverse neighborhood”) [114]—adding each new test observation one at a time and computing whether it is or not an outlier in comparison with the reference (i.e., training) data set. We also applied the KNN (k nearest neighbour) approach proposed by Sahigara et al. (2013) [34] and the method advanced by Roy et al. (2015) [33] using R code written in house.

### 4.7. Virtual Screening by QSAR

The 49 best-performing QSAR models were used to predict the activity of a data set consisting of 104,619 ZINC database compounds (the “named” subset, i.e., compounds that have names in the ZINC 15 database [115]). The 49 models were stacked using a simple majority voting for the decision; the performance of the stacking was assessed by applying the same majority voting to the independent predictions in the nested cross-validation loops. The compounds were ranked in decreasing order, from those predicted by 100% of the models to those predicted by only 51% of the models.

### 4.8. Molecular Docking Study

Crystallographic data available in the PDB database (PDB ID: 4MXO [116], PDB ID: 3QLG [117]) show that src-tyrosin kinase inhibitors engage the enzyme primarily at the hinge residues, a few amino acid residues having a particular relevance: Val 281, Ala 293, Met 314, Ile 336, Met 341, Leu 393 [118]. We intended to evaluate whether the molecules ranked in our virtual screening as active with highest confidence bind in the back pocket of the src-tyrosin kinase in a similar way with dasatinib or bosutinib. Docking was performed using AutoDock Vina [35] with default parameters under Yasara (version 19.7.20), and LeDock. Human c-src protein (PDB ID: 2src [119]) was used as a target. For Vina, the protein preparation was performed in Chimera (Resource for Biocomputing, Visualization, and Informatics at the University of California, San Francisco, CA, USA) using the Dock Prep module (deleting the ligand and water molecules, eliminating alternate locations of residues, replacing selenomethionine with methionine, etc.); protonation states were assigned with the addH module of Dock Prep, at physiological pH (about 7.4), using the default method. The active site for the Vina docking was defined as a cubic cell of 5 Å around the selected residues (mentioned above). The setup was performed with the YASARA molecular modeling software (YASARA Biosciences GmbH, Vienna, Austria), the compounds being sorted by the program by the free energy of binding (the best hit of 25 runs), this being used for post-analysis, as discussed below. For LeDock the protein preparation was carried out using the LePro module (with the default values) and the docking was run with the default values of the LeDock module; the binding pocket was also a rectangular box with a radius of 5 Å. Clustering by RMSD (1.0 Å) was used to reduce redundancy, and the score of the first cluster (obtained from 20 runs) was selected for each compound for post-analysis.

The SMILES structures corresponding to the ZINC codes of the compounds predicted as active in the virtual screening by at least 75% of the models were downloaded in Python with the help of the smilite package; they were then converted to sdf format in DataWarrior (adding 3D coordinates) and then to mol2 format (with hydrogens added) in Biovia Discovery Studio and batch split to individual mol2 files with Open Babel. Ligand energy minimization was performed with Marvin Sketch, v. 19.19. The mol2 files were used in the LeDock software (Lephar Research, Stockholm, Sweden) for virtual screening.

To estimate the performance of the docking a subset of the training set comprising 175 compounds (33 with ki < 20 nM, 67 with 500 < ki < 1000 nM, 32 with 1500 < ki < 2000 nM, and 43 compounds with ki > 10,000 nM) was used and “cutpointr” R package was employed to define the best cut-off point of computed energies of binding between actives and inactives, based on the sum of sensitivity and specificity. We also computed various ligand efficiency metrics, which have been reported in the literature to improve the docking scoring; they were computed by dividing the energy of binding to the molecular weight, number of heavy atoms, number of carbon atoms, partition coefficient, and Wiener index [120]. We also explored computing ligand efficiencies by dividing the energy of binding to the squared value of the partition coefficient, to the total surface area, McGowan volume, van der Waals volume from McGowan volume, and van der Waals volume from the Zhao–Abraham–Zissimos equation (metrics not reported previously). The “cutpointr” R package [121] was used to define the best cut-off point of computed energies of binding between active and inactive compounds, based on the sum of sensitivity and specificity. For further validation we also docked the co-crystallized ligand from the c-src protein (PDB ID 2csrc), namely the phosphoaminophosphonic acid-adenylate ester, and RMSD was computed for the first cluster of poses predicted by LeDock. RMSD computation was performed in R based on the well-known formula and the results were compared with those obtained with the online DockRMSD [122], the values obtained being identical. Following the strong suggestion of one of the reviewers of this paper, we tested the compounds predicted by both the QSAR models and docking to be active and evaluate their potential effects using the online version of the program PASS [38].

## 5. Conclusions

A total of 49 global QSAR models have been developed, predicting the c-src tyrosine kinase inhibition with reasonable accuracy (>70%) and positive predictive value (>70%). The 49 models were assembled by stacking and used for the virtual screening of over 100,000 named compounds from the ZINC database. Several hundreds of compounds were predicted to be active, depending on the decision threshold used. Those with the highest probability of being active were also subjected to molecular docking and for the majority (about 61%) of them the energies of binding obtained were consistent with a hypothesis of activity. External data from ChEMBL and PubChem confirmed that at least 7.83% (in the case of QSAR) or 6.67% (in the case of integrated QSAR and molecular docking) of the compounds are active on the c-src target; more than a quarter of the predictions were also confirmed by prediction performed by the online version of PASS The ratio of active compounds is smaller than what was to be expected from the nested cross-validation data, but still better than what one should expect from any high-throughput type of screening experiments.

## Figures and Tables

**Table 1:** Performance of the quantitative structure-activity relationship (QSAR) models selected.

Model *	BA (%)	PPV (%)	MMCE (%)	AUC (%)	TPR (%)	TNR (%)	Q_2_ − Q_2_, rnd
RF_anova_23	70.24	78.26	18.60	82.56	45.39	95.08	21.33
RF_auc_20	70.07	78.08	18.69	82.85	45.04	95.09	21.23
RF_cforest_13	70.07	79.39	18.60	82.96	44.80	95.34	21.33
RF_kruskal_30	70.52	77.42	18.60	82.61	46.35	94.68	21.33
RF_RFimp_30	71.54	80.04	17.73	86.03	47.69	95.39	22.19
RF_RF.SRCimp_20	71.01	77.44	18.31	83.76	47.18	94.83	21.62
RF_RF.SRCvarselect_10	72.93	78.72	17.34	86.01	51.29	94.56	22.58
RF_impurity_15	70.67	76.43	18.69	83.72	46.91	94.43	21.23
RF_permutation_10	71.53	80.51	17.83	83.63	47.86	95.20	22.10
RF_univariate_30	71.48	83.49	17.44	84.31	46.80	96.16	22.48
SVM_anova_30	71.83	71.26	19.07	82.08	51.60	92.05	20.48
SVM_auc_30	72.02	71.56	18.98	83.25	51.99	92.05	20.94
SVM_cforest_30	75.11	74.96	17.05	85.60	57.65	92.57	22.87
SVM_chi.sq_30	71.91	75.44	18.59	82.45	50.86	92.97	21.33
SVM_gainratio_30	72.03	72.78	18.98	82.85	51.99	92.07	20.94
SVM_information_30	72.44	73.34	18.59	83.91	52.54	92.35	21.33
SVM_kruskal_20	72.06	72.29	18.98	82.06	52.06	92.05	20.94
SVM_oneR_30	72.49	78.08	17.73	81.16	50.68	94.31	22.19
SVM_RFimp_30	74.74	74.71	17.25	86.92	57.16	92.32	22.68
SVM_RF.SRCimp_30	75.92	77.07	16.28	86.20	58.57	93.28	23.64
SVM_RF.SRCvarselect_20	76.33	76.22	16.28	86.75	60.10	92.56	23.64
SVM_impurity_30	73.96	73.86	17.82	84.27	55.61	92.30	22.10
SVM_permutation_20	72.14	73.82	18.59	84.37	51.58	92.71	21.33
SVM_relief_30	72.42	71.93	19.08	82.15	53.57	91.26	20.84
SVM_sym.uncertain_20	71.91	73.31	18.69	83.33	50.99	92.84	21.23
Adabm1_RFimp_30	71.06	73.50	19.08	83.49	49.11	93.00	20.84
Adabm1_RF.SRCvarselect_20	71.15	70.36	19.56	81.96	50.36	91.95	20.36
Adabm1_impurity_20	71.22	73.34	18.80	83.66	49.18	93.26	21.13
Adabm1_univariate_30	70.50	74.30	19.27	82.36	47.61	93.39	20.65
BartM_chi.sq_30	73.15	73.28	18.11	83.54	53.87	92.42	21.81
BartM_gainratio_20	71.61	70.19	19.37	82.45	51.57	91.64	20.56
BartM_information_20	73.56	73.52	17.92	84.08	54.68	92.44	22.00
BartM_RFimp_25	74.24	71.45	18.02	85.28	57.13	91.36	21.90
BartM_impurity_20	73.48	70.94	18.50	83.79	55.74	91.22	21.42
BartM_permutation_22	74.70	71.64	17.82	85.04	58.17	91.23	22.10
BartM_sym.uncertain_30	73.59	71.19	18.31	84.36	55.69	91.49	21.62
C50_anova_30	75.96	72.56	17.05	84.73	60.70	91.23	22.87
C50_auc_20	74.00	72.03	18.12	83.75	56.80	91.19	21.81
C50_cforest_20	75.08	71.62	17.73	85.06	59.32	90.84	22.19
C50_chi.sq_30	75.55	70.40	17.73	83.55	60.79	90.32	22.19
C50_gainratio_30	75.26	70.85	17.82	84.43	60.08	90.45	22.10
C50_kruskal_30	74.56	71.35	18.02	84.52	58.03	91.10	21.90
C50_oneR_30	73.91	72.78	18.41	83.62	57.06	90.76	21.52
C50_RFimp_30	78.56	75.39	15.32	87.24	65.23	91.89	24.60
C50_RF.SRCimp_30	76.21	72.82	17.05	85.45	61.32	91.10	22.87
C50_RF.SRCvarselect_20	77.64	72.08	16.76	87.84	65.43	89.86	23.16
C50_impurity_20	76.40	76.14	16.10	86.70	60.13	92.66	23.83
C50_permutation_30	75.93	72.28	16.96	86.29	60.51	91.36	22.96
C50_univariate_30	75.44	70.55	17.73	85.47	60.46	90.43	22.19

* Each model name is formed by three parts separated by an underscore: the first part of the name indicates the classifier, the second part the feature selection algorithm (in an abbreviated form), and the third part the number of features used to build the model. The names of the classification and feature selection algorithms are provided in Section 4. For instance, RF_anova_20 was a random forest based on features selected based on ANOVA (as implemented in “anova.test” within “mlr” R package) and the number of features used was 20. BA: balanced accuracy; PPV: positive predictive value; MMCE: mean misclassification error; AUC: area under the ROC curve; TPR: true positive rate; TNR: true negative rate; Q2 – accuracy; Q2, rnd - most probable random accuracy (as explained in the text).

**Table 2:** The most important molecular descriptors associated with the inhibition of the c-src tyrosine kinase.

Name	Interpretation	Descriptor Block (Group)	Frequency Occurring among the First Five Most Important Features
SpMax4_Bh(m)	Largest eigenvalue n. 4 of Burden matrix weighted by mass	Burden eigenvalues	14
DECC	Eccentric topological index	Topological indices	11
SpMax5_Bh(m)	Largest eigenvalue n. 5 of Burden matrix weighted by mass	Burden eigenvalues	8
SpMax3_Bh(m)	Largest eigenvalue n. 3 of Burden matrix weighted by mass	Burden eigenvalues	8
J_D	Balaban-like index from topological distance matrix (Balaban distance connectivity index)	2D matrix-based descriptors	6
F06[C–N]	Frequency of C–N at topological distance 6	2D Atom Pairs	5
Chi1_EA(dm)	Connectivity-like index of order 1 from edge adjacency mat. weighted by dipole moment	Edge adjacency indices	4
P_VSA_MR_6	P_VSA-like on Molar Refractivity, bin 6	P_VSA-like descriptors	3
SpMax6_Bh(m)	largest eigenvalue n. 6 of Burden matrix weighted by mass	Burden eigenvalues	3
N-073	Ar2NH/Ar3N/Ar2N-Al/R..N..R	Atom-centered fragments	2
F05[C–N]	Frequency of C–N at topological distance 5	2D Atom Pairs	2

A total of 19 other descriptors occurred only once among the five most important features identified by each of the 17 feature selection algorithms.

**Table 3:** Compounds predicted to be active by both the assembled QSAR models and ligand docking.

ZINC Code	Substance Name	Confirmation in Wet Lab Experiments *	Activity Confirmed on Other Tyrosin Kinases *	Presence in the Training Set	Energy of Binding **
ZINC000001550477	Lapatinib	Yes	Yes	Yes	−10.07 (0.67)
ZINC000034638188	Pf-562271	Yes	Yes	Yes	−9.3 (0.74)
ZINC000063298074	Ilorasertib	Yes	Yes	Yes	−10.09 (0.66)
ZINC000034800096	Gw583373a	No	Yes	No	−11.02 (1.01)
ZINC000027184814	Vibriobactin	NA	No	No	−9.77 (0.74)
ZINC000034800093	Gw580496a	No	Yes	No	−9.33 (1.09)
ZINC000150528975	Vedroprevir	NA	No	No	−11.51 (1.04)
ZINC000034800112	Gw576484x	No	Yes	No	−10.36 (0.84)
ZINC000072190218	Avatrombopag	NA	No	No	−9.28 (0.43)
ZINC000034800091	Gw576609a	No	Yes	No	−11.38 (0.69)
ZINC000044418656	Gw784684x	No	Yes	No	−10.77 (0.93)
ZINC000042804069	Gsk-182497a	No	Yes	No	−9.57 (0.37)
ZINC000103297739	Defactinib	No	Yes	No	−10.23 (0.40)
ZINC000004215255	Cefpimizole	NA	No	No	−10.54 (0.70)
ZINC000042834127	Gsk1751853a	No	Yes	No	−10.34 (1.40)
ZINC000014945166	Gw830365a	No	Yes	No	−9.53 (0.29)
ZINC000150339466	Ciluprevir	NA	No	No	−10.95 (0.88)
ZINC000043195317	Golvatinib	No	Yes	No	−14 (1.06)
ZINC000042201866	Gw566221a	No	Yes	No	−10.06 (0.71)
ZINC000095615094	Patellamide G	NA	No	No	−9.32 (0.79)
ZINC000003604326	Vaneprim	NA	No	No	−11.01 (0.79)
ZINC000002007399	Gw458787a	No	Yes	No	−10.95 (0.76)
ZINC000028639340	Posaconazole	NA	No	No	−10.92 (1.01)
ZINC000072122048	Gsk259178a	No	Yes	No	−12.44 (0.49)
ZINC000068204830	Daclatasvir	NA	No	No	−10.75 (0.42)
ZINC000043131420	Fostamatinib	NA	Yes	No	−10.77 (1.11)
ZINC000169289453	Simeprevir	NA	No	No	−11.45 (0.88)
ZINC000042834162	Gw869810x	No	Yes	No	−12.11 (0.76)
ZINC000049709569	Asperazine	NA	No	No	−11.6 (0.82)
ZINC000096928979	Deleobuvir	NA	No	No	−10.2 (0.68)
ZINC000042201868	Gw568377a	No	No	No	−9.36 (0.60)
ZINC000014945147	Gw809897x	Yes	Yes	No	−10.44 (0.71)
ZINC000014945171	Gw830263a	Yes	Yes	No	−10.53 (0.57)
ZINC000014945045	Gw569530a	No	Yes	No	−9.52 (0.55)
ZINC000003925087	Gw806742x	Yes	Yes	No	−10.43 (0.78)
ZINC000095618748	Candesartan O-Glucuronide	NA	No	No	−9.71 (0.58)
ZINC000098052868	Olcegepant	NA	No	No	−9.55 (0.48)
ZINC000049833405	Preulicyclamide	NA	No	No	−11.13 (0.62)
ZINC000034800110	Gw574782a	No	Yes	No	−10.42 (0.60)
ZINC000014965596	Gw683134a	Yes	Yes	No	−10.91 (0.80)
ZINC000034800112	Gw576484x	No	Yes	No	−9.93 (0.36)
ZINC000019862646	Fedratinib	Yes	Yes	No	−10.23 (0.64)
ZINC000150377731	Bms-247243	NA	No	No	−10.42 (0.83)
ZINC000003986669	Bx-795	Yes	Yes	No	−9.28 (0.69)
ZINC000095615898	Tyrokeradine A	NA	No	No	−11.14 (0.76)
ZINC000003919988	L-766892	NA	No	No	−9.59 (0.67)
ZINC000095544067	Ulithiacyclamide F	NA	No	No	−9.76 (0.52)
ZINC000049889335	Edulirin A	NA	No	No	−11.45 (1.04)
ZINC000003995140	Gw621823a	No	Yes	No	−10.63 (0.63)
ZINC000040379218	Gw684626b	No	Yes	No	−10.46 (0.87)
ZINC000034800121	Gw567808a	No	Yes	No	−10.42 (0.53)
ZINC000169306513	Hydroxyitraconazole	NA	No	No	−9.78 (1.02)
ZINC000169368380	Kni-1039	NA	No	No	−10.13 (0.41)
ZINC000150601177	Ombitasvir	NA	No	No	−10.07 (0.69)
ZINC000040404350	Gsk-969786a	No	Yes	No	−10.2 (0.75)
ZINC000150592451	Micromide	NA	No	No	−12.96 (1.00)
ZINC000028249631	Pd-170292	NA	No	No	−10.1 (0.73)
ZINC000169366333	Porphyrin	NA	No	No	−11.05 (0.71)
ZINC000034800119	Gw576924a	No	Yes	No	−10.18 (0.92)
ZINC000150362888	Pyropheophytin B	NA	No	No	−10.23 (0.73)
ZINC000100057121	Tegobuvir	NA	No	No	−10.55 (0.58)
ZINC000103213128	Heptamethylene 1,7-Bis-Imadacloprid	NA	No	No	−9.58 (0.47)
ZINC000169291993	Sansanmycin F	NA	No	No	−9.5 (0.56)
ZINC000230052516	Urobilin	NA	No	No	−10.9 (0.85)
ZINC000003994828	Brecanavir	NA	No	No	−10.41 (0.86)
ZINC000169363931	Ansacarbamitocin C	NA	No	No	−10.56 (0.52)
ZINC000095535868	Rwj-58259	NA	No	No	−10.09 (0.77)
ZINC000003921862	Tallimustine	NA	No	No	−9.76 (0.67)
ZINC000063933734	Rebastinib	No	Yes	No	−9.73 (0.57)
ZINC000095615652	Patellamide C	NA	No	No	−9.46 (0.73)
ZINC000197688172	S-[(3e,5z)-3,5-Octadienoate	NA	No	No	−9.6 (0.67)
ZINC000014965588	Gw709042a	No	Yes	No	−9.89 (0.89)
ZINC000085537136	Barixibat	NA	No	No	−9.72 (0.56)
ZINC000169291499	Kibdelomycin	NA	No	No	−10.99 (0.66)
ZINC000003946578	Mitratapide	NA	No	No	−10.41 (0.62)
ZINC000001481922	Setipafant	NA	No	No	−10.05 (0.62)
ZINC000072173092	Deoxyvobstusine Lactone	NA	No	No	−9.66 (0.64)
ZINC000006717126	Quarfloxin	NA	No	No	−9.85 (0.78)
ZINC000077301904	Losartan N2-Glucuronide	NA	No	No	−10.86 (1.27)
ZINC000150609364	Pseudoceratinazole A	NA	No	No	−11.38 (0.97)
ZINC000095616246	Ulithiacyclamide E	NA	No	No	−9.35 (0.69)
ZINC000068151111	Narlaprevir	NA	No	No	−9.96 (0.44)
ZINC000150351429	Phytosulfokine B	NA	No	No	−9.7 (0.70)
ZINC000003989268	Ceftaroline Fosamil	NA	No	No	−9.84 (0.62)
ZINC000008552132	Stafac	NA	No	No	−11.01 (0.91)
ZINC000095618880	Clofazimine Glucuronide	NA	No	No	−9.65 (0.58)
ZINC000096006065	Xv638	NA	No	No	−9.56 (0.57)
ZINC000169292535	Rifapentine	NA	No	No	−12.81 (0.92)
ZINC000150341961	Mafodotin	NA	No	No	−9.32 (0.71)

* Based on ChEMBL and PubChem data for each substance (“Yes” means that there are at least limited confirmatory data in one of the public databases, “No” means that there is no such confirmatory data; NA—data not available at all). ** For an estimation of the docking error we provided in brackets the standard deviation of the energy of binding computed from the value of the different clusters of 20 poses.

## Supplementary Materials

Supplementary Materials can be found at https://www.mdpi.com/1422-0067/21/1/19/s1.

## Abbreviations

QSAR	Quantitative structure-activity relationship
AUC	Area under the ROC curve
PPV	Positive predictive value
RMSD	Root-mean-square deviation
BA	Balanced accuracy
MMCE	Mean misclassification error
TPR	True positive rate
TNR	True negative rate
KDEOS	Kernel Density Estimation Outlier Score
INFLO	Influenced Outlierness
MAO-A	Monoamine oxidase A
RF	Random forests
SVM	Support vector machines
BART	Bayesian additive regression trees
