# Supplementary material for: Use of QSAR Global Models and Molecular Docking for Developing New Inhibitors of c-src Tyrosine Kinase
Source: Int J Mol Sci. 2019 Dec 18;21(1):19. doi: 10.3390/ijms21010019 (PMC6981969; doi:10.3390/ijms21010019)
Supplement: Supplementary file 1 [file ijms-21-00019-s001.zip › Paper_supplementary_files_v_2/Formulae for the computing of performance metrics.docx]

Formulae for the computing of performance metrics:

**TPR:** $\frac{TP}{TP+FN,}$ where TP = number of true positive samples, and FN = number of false negative samples

**TNR:** $\frac{TN}{FP+TN,}$ where TT = number of true negative samples, and FP = number of false positive samples.

**Balanced accuracy (BA):** $\frac{TPR+TNR}{2}$

**Positive Predictive Value (PPV):** $\frac{TPR}{TPR+FPR}$

**Mean misclassification error rate:** mean (prediction != truth)

**AUC:** an algorithm for the computation of this more complex metric is available in Tharwat, A. Classification assessment methods. Applied Computing and Informatics 2018 (<https://doi.org/10.1016/j.aci.2018.08.003>) (Open Access).

Q2: $\frac{TP+TN}{TP+TN+FP+FN}$
