# Supplementary figures and images for: Use of QSAR Global Models and Molecular Docking for Developing New Inhibitors of c-src Tyrosine Kinase
Source: Int J Mol Sci. 2019 Dec 18;21(1):19. doi: 10.3390/ijms21010019 (PMC6981969; doi:10.3390/ijms21010019)

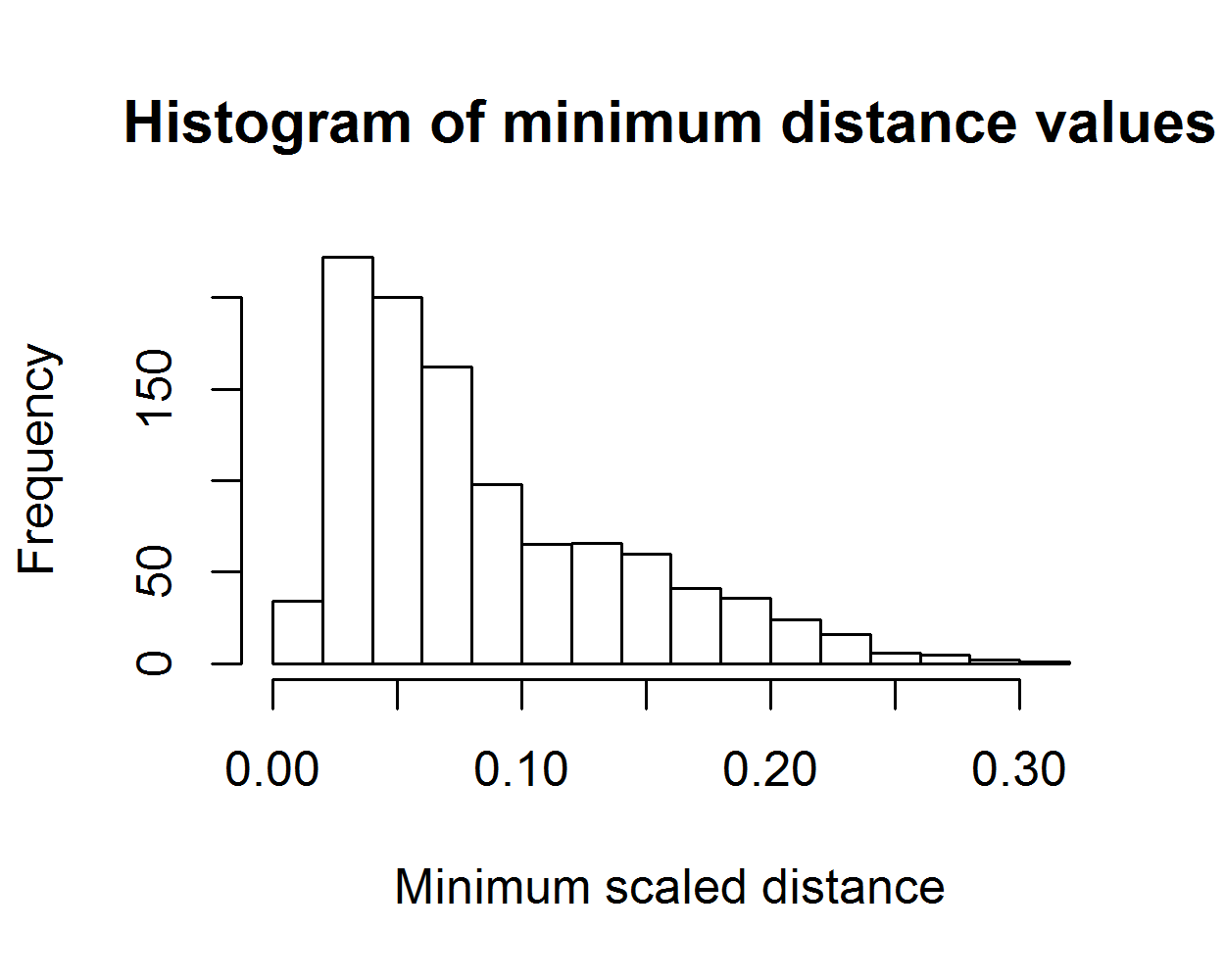

Supplement: Supplementary file 1 [file ijms-21-00019-s001.zip › Paper_supplementary_files_v_2/S1Fig_minimum_distance_values.tif]

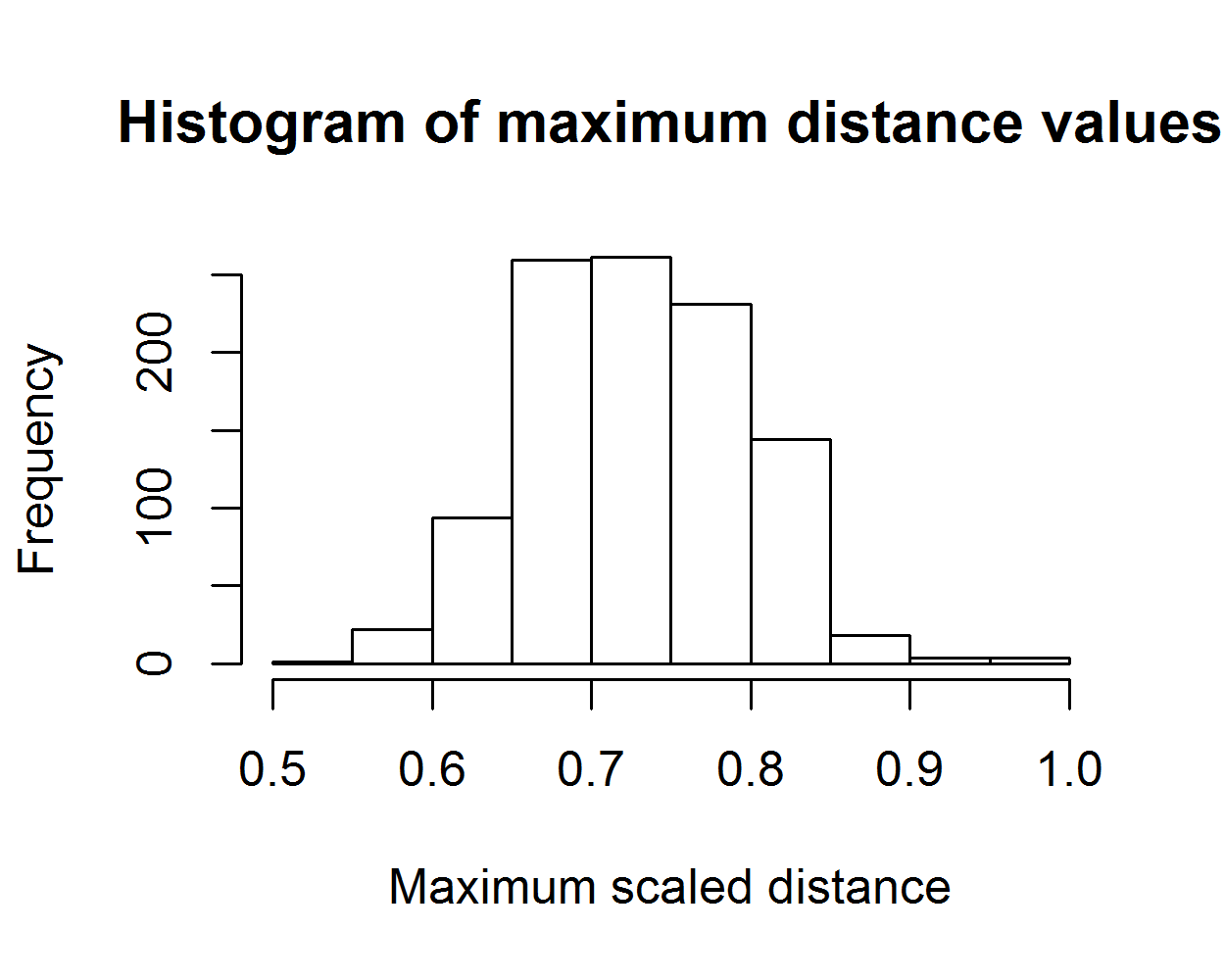

Supplement: Supplementary file 1 [file ijms-21-00019-s001.zip › Paper_supplementary_files_v_2/S2Fig_maximum_distance_values.tif]

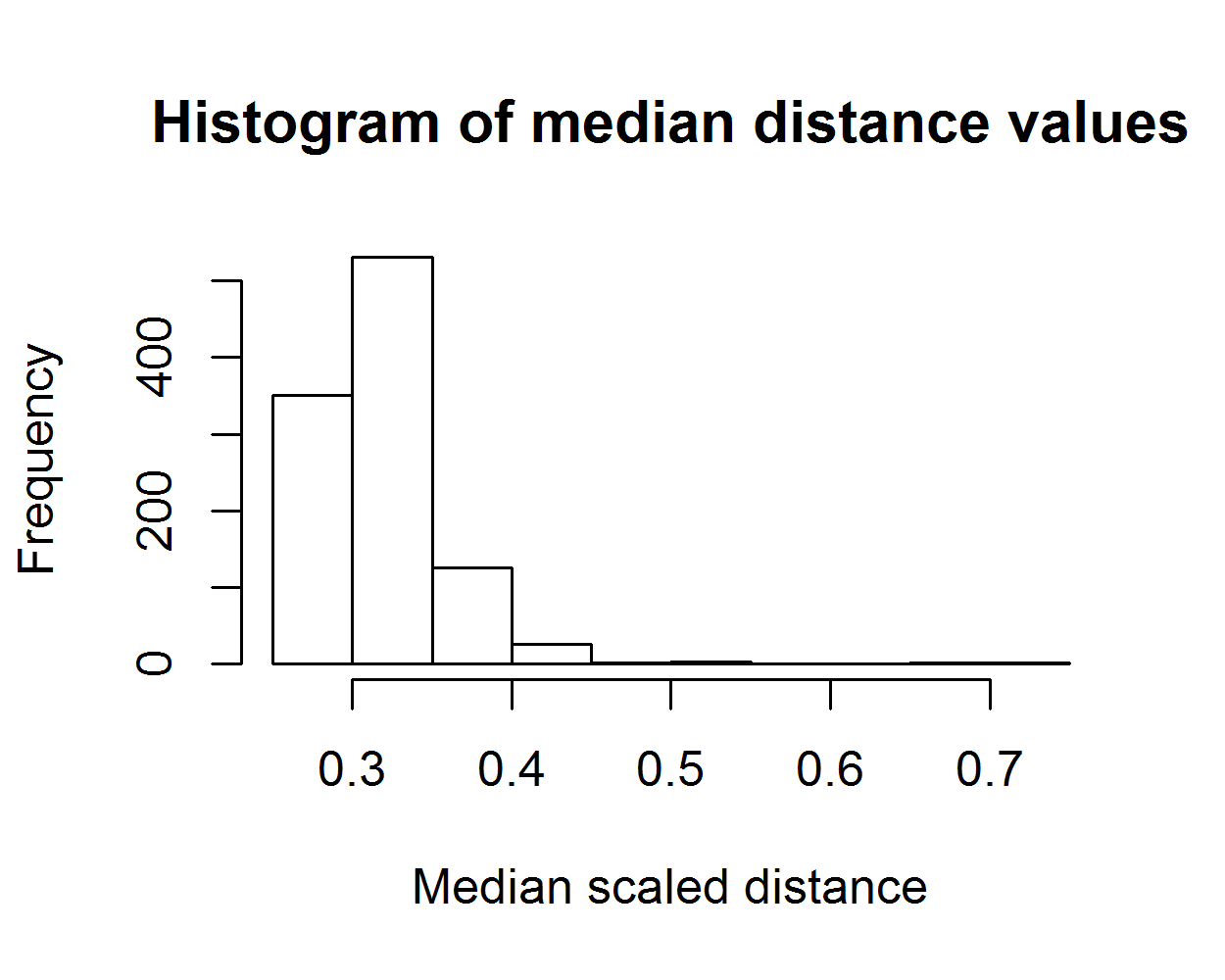

Supplement: Supplementary file 1 [file ijms-21-00019-s001.zip › Paper_supplementary_files_v_2/S3Fig_median_distance_values.tif]
